# Survey of clinical recalls from breast screening at Nottingham Breast Institute: implications for practice

**DOI:** 10.1186/bcr3291

**Published:** 2012-11-09

**Authors:** MZ Mvere, S Tennant

**Affiliations:** 1 Nottingham Breast Institute, Nottingham, UK

## Introduction

In our unit, symptomatic women identified at the time of screening (that is, those reporting a breast symptom, or in whom the radiographer notices a breast sign) are recalled for further assessment despite a normal mammogram. Several studies have suggested that the cancer detection rate in this group of patients is low. The aims of this survey were therefore to determine the cancer yield and whether our current practice of recalling women on the basis of clinical history alone is a worthwhile practice.

## Methods

The total number of women screened, and those recalled to assessment, during a 24-month period were retrospectively identified using NBSS. Women recalled on the basis of clinical history alone were identified and information about the screening assessment clinic visit was reviewed.

## Results

Of the 45,940 women screened during this time period, 337 cancers (invasive and DCIS) were diagnosed (7.3 cancers/1,000 women screened). A total of 335 cancers were diagnosed in the 1,177 women recalled because of an abnormal mammogram (28.4%). Two cancers were detected in the 116 women recalled for clinical history alone (1.72%). The cancer detection rate is therefore 17 times lower in the clinical recall group than in those called for a mammographic abnormality.

## Conclusion

The cancer detection rate in women recalled on the basis of clinical history alone is low and, given the extra time and resources required in assessing these women, our current practice should be reviewed.

